# Docetaxel chemotherapy plus androgen-deprivation therapy in high-volume disease metastatic hormone-sensitive prostate cancer in Chinese patients: an efficacy and safety analysis

**DOI:** 10.1186/s40001-022-00773-1

**Published:** 2022-08-11

**Authors:** Zhuifeng Guo, Xuwei Lu, Fan Yang, Liang Qin, Ning Yang, Jiawen Wu, Hang Wang

**Affiliations:** 1 Department of Urology, Minhang Hospital, Fudan University, Shanghai, China; 2 Department of Urology, Zhongshan Hospital, Fudan University, Shanghai, China

**Keywords:** Prostate cancer, High-volume disease, Metastatic, Hormone-sensitive, Docetaxel, Androgen-deprivation therapy, Chemotherapy

## Abstract

**Objective:**

To investigate the efficacy and safety of docetaxel chemotherapy combined with androgen-deprivation therapy for patients with high-volume disease metastatic hormone-sensitive prostate cancer.

**Methods:**

153 cases of high-volume disease metastatic hormone-sensitive prostate cancer in Minhang Hospital between January 2018 and December 2019 were analyzed retrospectively, including the number of patients, age, initial PSA level, Gleason score, TNM stage and ECOG score. 90 patients in the endocrine therapy group received continuous ADT, and 63 patients in the combined chemotherapy group received docetaxel plus ADT. The progression-free survival time (time from initiation of prostate cancer treatment to progression to CRPC), PSA response rate, and adverse reactions were compared between the two groups.

**Results:**

All 153 cases were closely followed up for a period of 12.3–35.3 months, with a median follow-up time of 23.5 months. The median time to reach the lowest point of PSA in the two groups was 6.3 months and 7.9 months (*P* = 0.018) in the combination chemotherapy group and the ADT group alone, with 27 (42.9%) and 12 (13.3%) cases in the two groups Within 12 months of treatment, PSA decreased to below 0.2 ng/ml (*P* = 0.02), and progression-free survival was 16.9 months (6.5–28.5 months) and 11.2 months (4.3–22.7 months) in the two groups. (*P* < 0.001). There were 18 cases (28.6%) and 54 cases (60%) in the two groups with disease progression (*P* < 0.001). There were 6 cases (9.5%) and 15 cases (16.7%) in the combination chemotherapy group and the ADT group died of prostate cancer and related complications, respectively. All 63 cases in the combined chemotherapy group completed 6 cycles of chemotherapy. 39 (61.9%) cases experienced varying degrees of neutropenia, of which 12 (19%) experienced grade 3–4 neutropenia, with 6 cases (9.5%) developed febrile neutropenia. 30 cases (47.6%) had toxic reactions in the digestive system, and 3 case (4.3%) had grade 3 liver dysfunction. 27 cases (42.8%) had skin and mucosal toxicity. 9 cases (14.3%) had mild fluid retention. No blood and digestive toxicity were observed in the ADT group. 33 cases (52.4%) and 48 (53.3%) of the two groups had symptoms of afternoon hot flashes and fatigue, (*P* = 0.961).

**Conclusion:**

Docetaxel chemotherapy combined with endocrine therapy could be one of effective treatments for delaying castration resistance of HVD-mHSPC, which could prolong PFS effectively and obtain a higher PSA response rate, high safety under close monitoring, and controllable adverse reactions.

## Introduction

Prostate cancer is the second most common cancer in the world, and is the fifth leading cause of cancer-related death in males. It was estimated that there were 248,000 new prostate cancer cases and about 69,000 prostate cancer-related deaths in the USA in 2021 [1]. Because of regional differences in prostate-specific antigen (PSA) monitoring, the proportion of newly diagnosed patients with metastatic prostate cancer is still high in China [2]. The higher risk of metastases associated with higher Gleason grade, PSA velocity, and characteristics on multiparametric magnetic resonance imaging [3] and genomic alterations in aggressive prostate cancer manifested early, many years before the metastatic disease was detected clinically [4]. The latest subgroup analysis of STAMPEDE arm C [5] and CHAARTED [6] proved that high-volume disease metastatic prostate cancer (HVD-mHSPC) significantly improves after docetaxel (DTX) chemotherapy plus androgen-deprivation therapy (ADT). However, there are very limited data of DTX plus ADT in HVD-mHSPC patients in China. The data presented here analyze the safety and efficacy of DTX chemotherapy for 153 HVD-mHSPC patients from January 2018 to December 2019 to help clinicians make reasonable clinical decisions, prolong the survival time of HVD-mHSPC patients and improve the quality of life.

## Patients and method

### Baseline characteristics

All 153 patients included came from new cases in the specialized disease outpatient departments of Minhang Hospital. All patients signed informed consent for treatment. A total of 63 patients received 3-week DTX chemotherapy plus ADT (DTX group) and 90 patients received ADT alone (ADT group). General data from the two groups include the patient’s age, newly treated PSA level and Gleason score (GS), clinical TNM classification (cTNM) and ECOG score of physical condition (Table 1). All patients were diagnosed with multiple bone metastases, regional or distant lymph node metastasis and visceral metastasis as determined by PET–CT, isotope whole-body bone scan and pelvic enhanced MRI. Inclusion criteria were patients with newly diagnosed HVD-mHSPC and the definition of HVD-mHSPC includes: 1. Endocrine therapy sensitivity: serum testosterone from the prostate showed a decrease of PSA or imaging remission after castration during ADT treatment (< 1.7 nmol/L). 2. High-volume disease: visceral metastasis and/or ≥ 4 bone metastases, including at least one bone metastasis outside the pelvis or spine [6]. Exclusion criteria were patients with metastatic recurrence after prior local curative treatment, previous local or systemic therapy for prostate cancer.

**Table 1:** Comparison of general data of the combined DTX group and the ADT group

Variable	DTX + ADT	ADT	*P*
Number of patients	63	90	
Age, years, median (range)	69 (62–79)	77.5 (66–91)	*0.001
Age, years, *n* (%)			
< 70	33 (52.4)	15 (16.7)	
≥ 70	30 (47.6)	75 (83.3)	*0.001
PSA level, ng/ml,	226.3	146.2	
Median (range)	(29.4–2892)	(35.3–2238)	*0.024
GS, *n* (%)			
6–7	27 (42.9)	30 (33.3)	
8–10	36 (57.1)	60 (66.7)	0.303
T stage, *n* (%)			
1–2	18 (28.6)	27 (30)	
3–4	45 (71.4)	63 (70)	0.991
N stage, *n* (%)			
N0	18 (28.6)	21 (23.3)	
N1	45 (71.4)	69 (76.7)	0.587
M stage, *n* (%)			
M1a	0 (0)	0 (0)	
M1b	51 (80.9)	72 (80.0)	
M1c	12 (19.1)	18 (20.0)	0.951
ECOG, *n* (%)			
0	30 (60.0)	12 (13.3)	
1	24 (26.7)	27 (30.0)	
2	9 (13.3)	24 (26.7)	
3	0 (0)	27 (30.0)	*0.001

*Statistically different

### Treatment characteristics

All patients received continuous ADT (LHRH-α, a luteinizing hormone-releasing hormone analogue, injected subcutaneously once a month or castrated surgically). During ADT, the serum testosterone from all patients is reduced to castration level (< 1.7 mmol/L). 63 patients in the DTX group were treated with ADT for 1–2 months [7] before DTX chemotherapy. The chemotherapy scheme was DTX 75 mg/m^2^ via intravenous drip on the first day of every 21-day cycle, and combined with oral dexamethasone 7.5 mg 12 h, 3 h and 1 h before DTX treatment. A total of 6 cycles were completed in the overall treatment. PSA was detected every 3 weeks during chemotherapy, and every month after treatment as a regular follow-up for both groups. Patients from the two groups underwent imaging evaluation by PET–CT, isotope whole-body bone scan and pelvic enhanced MRI and re-examination every 3 months during the treatment [8]. Whether or not the patient underwent the imaging progress was determined using the evaluation standard for solid tumors (RECIST 2.0 standard). PSA progression is defined as the increase of PSA for three consecutive times at intervals of one week or more, and the increase of PSA for two consecutive times is over 50% greater than the lowest value with PSA > 2 ng/ml. PSA reaction is defined as a detection of 2 consecutive PSA < 0.2 ng/ml readings, with at least a 4-week interval [9].

Primary end point of the study was progression-free survival (PFS), which is the time from initial treatment of prostate cancer to the progression to CRPC where PSA continues to rise and/or imaging progress occurs after serum testosterone is maintained at castration level. Other observation indexes include PSA remission rate and various adverse reactions according CTCAE standards.

### Statistical analysis

All statistical analyses were performed using the Statistical Package for the Social Sciences (SPSS, version 22; SPSS Inc., IBM Corp., Armonk, NY, USA). Baseline characteristics were compared using either the Chi-squared or Student’s *t*-test. Non-normal distribution data were represented by M(Q_R_), and count data were represented by frequency and percentage. Time to progression was calculated using Kaplan–Meier plots and the log-rank test. The difference was statistically significant when *P* < 0.05.

## Results

### Efficacy

**Fig. 1:**
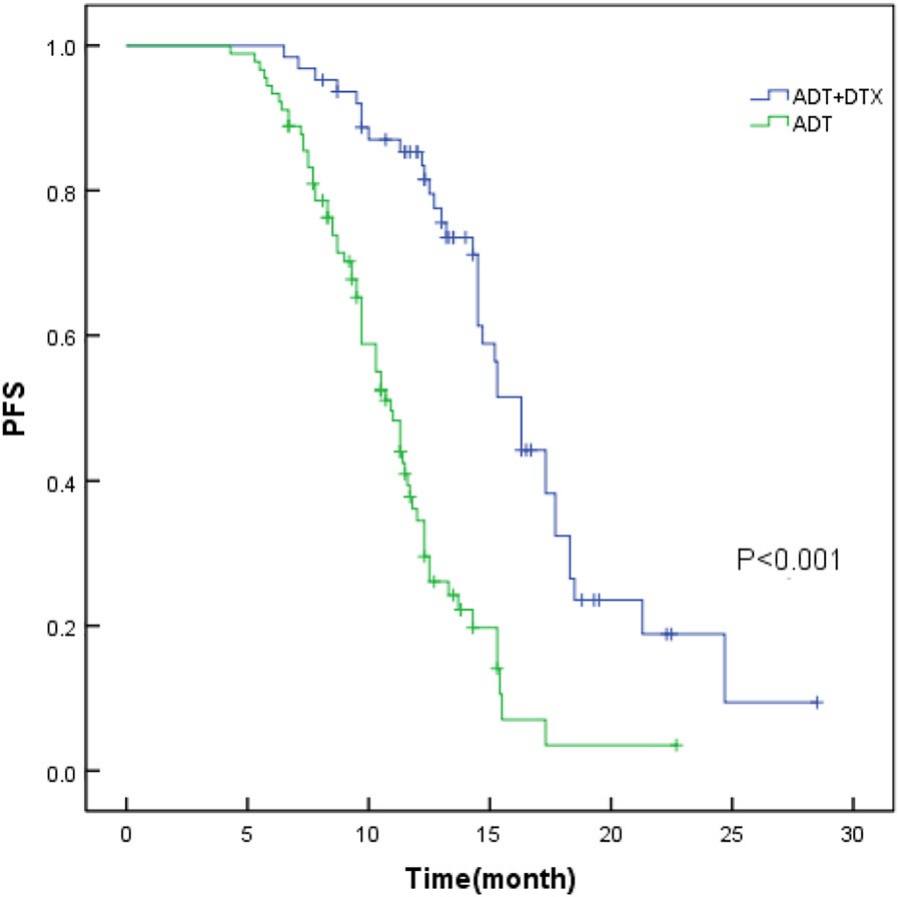
A comparison of PFS curves of patients in the combined DTX group and the ADT group

153 patients were tracked closely for 12.3–35.3 months, with a median of 23.5 months. In the initial stage of treatment, PSA decreased in both groups, and the median time to PSA nadir was 6.3 months in the combined DTX group, and 7.9 months in the ADT group (*P* = 0.018). Patients who received chemotherapy were more likely to have a nadir value of PSA < 0.2 ng/ml compared to ADT alone [27 patients (42.8%) and 12 patients (13.3%), respectively, *P* < 0.001]. The median OS was not reached in either group. There was some disparity between disease progression in both groups, 18 cases (28.6%) in the combined DTX group, and 54 cases (60.0%) in the ADT group (*P* < 0.001). There was a significant improvement in biochemical PFS in the chemotherapy plus ADT group (16.9 vs 11.2 months, *P* < 0.001) (Fig. 1). There were 6 deaths (9.5%) in the cohort of patients receiving DTX and 15 deaths (16.7%) in the ADT group due to prostate cancer progression or other related complications.

### Safety

All 63 patients in the DTX group completed 6 cycles of chemotherapy, of whom, 9 (14.3%) had delayed chemotherapy due to adverse reactions, 6 (9.5%) had febrile neutropenia (FN), and 3 (4.8%) had grade 3 hepatic insufficiency. The most common adverse reaction in the DTX group was hematological toxicity. In total, 6 patients (9.5%) needed to be hospitalized during the course of chemotherapy due to FN, all of whom were subsequently treated with antibiotics and G-CSF before neutrophils recovered to more than 1.5 × 10^9^/L. Other various adverse reactions are shown in Table 2. 33 patients (52.4%) in the combined DTX group and 48 patients (53.3%) in the ADT group suffered the symptoms of hot flashes and fatigue (*P* = 0.961).

**Table 2:** Related adverse reactions of ADT combined with DTX chemotherapy

Adverse events	number(%)
Hematologic toxicity	
Grade I–II neutropenia	27 (42.8)
Grade III–IV neutropenia	6 (9.5)
Febrile neutropenia, FN	6 (9.5)
Digestive system toxicity	
Nausea or vomiting	18 (28.6)
Defecation action changes	9 (14.3)
Grade III hypohepatia	3 (4.8)
Skin mucosal toxicity	
Hair loss	21 (33.3)
Skin rash or itching	6 (9.5)
Fluid retention	
Mild edema of limbs	9 (14.3)

## Discussion

Most patients with metastatic prostate cancer (mPC) initially responded positively to androgen suppression, however, invariably became resistant to hormone manipulation after 18–24 months from treatment [10]. Therefore, it is important to study effective treatments to delay the progress of mHSPC to mCRPC.

At present, a number of foreign research results confirm that mHSPC benefits from DTX combined with ADT, especially in the HVD-mHSPC population. GETUG-AFU15 was the first randomized controlled trial to evaluate the use of DTX in the treatment of mHSPC patients. The results showed that the combined DTX group had a 10-month extension of bPFS (22.9 months vs 12.9 months, *P* < 0.001), and the high-volume disease group had a 6-month extension of bPFS (15.2 months vs 9.2 months, *P* < 0.001) [11]. Sub-group analysis of CHAARTED showed that patients with high-volume disease benefited more obviously from treatment, the median OS increased by 17 months (49.2 months vs 32.2 months, *P* < 0.001) and the median time to CRPC was prolonged by 8.5 months (20.2 months vs 11.7 months, *P* < 0.001) [6]. Compared with ADT alone, the median OS of the DTX group was prolonged by 15 months (60 months vs 45 months, *P* < 0.001), and FFS was prolonged by 17 months (37 months vs 20 months, *P* < 0.001) in subgroup analysis of STAMPEDE [5]. All the results confirm that ADT combined with DTX chemotherapy significantly prolongs the overall survival of patients with mHSPC, especially those with high-volume disease. At present, there are very few reports about DTX chemotherapy combined with ADT used to treat HVD-mHSPC patients in China. The latest prospective single-arm, single-center phase II clinical study [9] showed that the PSA response rate was 30.9% at 6 months and 25.5% at 12 months in patients who underwent combined DTX treatment. The 1-year PFS rate was 66.5%, and the median OS and PFS was not reached. In our study, although patients in the combined DTX group had advantages in age and physical strength, there was no significant difference in Gleason score and TNM stage between the two groups. The results showed that PFS in the combined DTX group was 5.7 months longer than that of the ADT group. This is basically consistent with the extension of PFS by 6 months and 8.5 months in the GETUG-AFU15 and CHAARTED high-volume disease subgroup analyses [12, 13]. When comparing the GETUG-AFU15 and CHAARTED trials, which were prospective randomized controlled trials with uniform inclusion criteria, there was no significant difference in baseline level. However, in this study, considering the difference in patients’ tolerance to chemotherapy, there were differences in baseline levels between the groups. There were similar real-world retrospective studies conducted abroad where the baseline levels were also different and the median age was 68 and 81 years (*P* = 0.001) [7]. A higher PSA reaction rate indicates higher OS in the treatment of prostate cancer [14]. The newly diagnosed PSA level of the combined DTX group was higher, indicating that tumor load was higher. However, the time taken to reach PSA nadir was shorter than that of the ADT group, indicating that the combined DTX group can benefit even in high levels of PSA.

The most common adverse reaction of DTX was hematological toxicity, and FN accounted for 33% of delays in this therapeutic method. According to other research statistics, 17% of patients with DTX in mHSPC had grade 3–4 FN [7]. 12 cases (19%) had grade 3–4 neutropenia, and 6 cases (9.5%) had FN, all of whom recovered after timely G-CSF treatment. As the median time for neutrophils to reach nadir after DTX administration is 7 days, this interval can be shortened in patients undergoing multiple treatments [8]. FN patients were prone to life-threatening infections. This should be closely monitored with care.

Other adverse reactions caused by DTX mainly include allergic reaction, fluid retention, neurotoxicity, skin toxicity, liver function damage and fatigue [15]. In this study, three patients delayed chemotherapy due to grade 3 hepatic insufficiency. This occurred because DTX was being metabolized through the liver, causing increased liver load during the combined endocrine drug treatment. Other adverse reactions were gradually relieved by close follow-up, symptomatic treatment and appropriate psychological intervention.

As both groups were treated with continuous ADT, there were varying degrees of hot flashes and fatigue. There was no significant difference between the two groups, and no special intervention measures were required. Although there were no bone-related events in the study, more than half of the patients suffered from high-volume bone metastasis. The risk of osteoporosis based on ADT treatment should be minimized. It was recommended that calcium 1200 mg/day and vitamin D 800–1000 IU/day be supplemented routinely, and bisphosphate should be given when necessary [16].

In conclusion, the safety and efficacy of first-line DTX in HVD-mCRPC shown in the this study are comparable to some previous clinical trials. However, the fact that this study is a single-center retrospective study should be noted as a limitation. Patients in the combined DTX group had favorable age, physical fitness and tolerance to treatment than those in the simple ADT group. The overall number of cases in this study is small, and the long-term follow-up results to confirm any OS benefits have not yet been completed. Patients progressed to mCRPC were treated with different novel hormonal therapies including abiraterone, apalutamide or enzalutamide, etc., it was not possible to determine whether total OS benefited from docetaxel treatment, which was an inadequacy of the study. The real-world data of Chinese HVD-mHSPC patients undergoing ADT combined with DTX are very limited. While referring to foreign data for clinical practice, analyzing relevant data from the Chinese population in time will better guide clinical practice in the future. We still need to further carry out prospective randomized controlled clinical research with multi-centers, increase the number of research subjects and reduce the selection bias to improve the safety of applying ADT combined with DTX in HVD-mHSPC patients.

## Data Availability

The data-sets used and/or analyzed during the current study available from the corresponding author on reasonable request.
